# Global Insights on Wearable Technology Adoption by Coaches: Determinants of Current Use, Decision Making, and Future Intention To Use

**DOI:** 10.1186/s40798-025-00919-5

**Published:** 2025-11-21

**Authors:** Peter Düking, André Forster, Pamela Wicker, Bas Van Hooren, Lukas Masur, Michele Zanini, Billy Sperlich

**Affiliations:** 1 Department of Sports Science and Movement Pedagogy, Technische Universität Braunschweig, Braunschweig, Germany; 2 Differential Psychology, Personality Psychology and Diagnostics, Department of Psychology, University Würzburg, Würzburg, Germany; 3 Chair for Sport and Society, Department of Sports Science, Bielefeld University, Bielefeld, Germany; 4 NUTRIM Institute of Nutrition and Translational Research in Metabolism, Department of Nutrition and Movement Sciences, Maastricht University Medical Centre+, Maastricht, The Netherlands; 5 Integrative and Experimental Exercise Science, Department of Sport Science, University of Würzburg, Würzburg, Germany; 6 School of Sport, Exercise, and Health Sciences Loughborough University, Loughborough, UK; 7 School of Education, Childhood, Youth and Sport, The Open University, Milton Keynes, United Kingdom; 8 Italian Triathlon Federation, Rome, Italy

**Keywords:** Data-informed training, Digital health, Digital training, EHealth, Innovation, Artificial intelligence, MHealth

## Abstract

**Background:**

This study examines whether coaches use wearable technologies to individualize training procedures, and which factors influence both their current use and future intention to use such devices.

**Methods:**

Based on the technology acceptance model, we developed a questionnaire to assess the use of wearable technology for individualizing training procedures. Following a pilot investigation that included an exploratory analysis of a sample of 36 coaches, multiple regression models were used to confirm these exploratory results in a larger sample of 130 coaches (*n* = 5 Tier 1, *n* = 47 Tier 2, *n* = 52 Tier 3, *n* = 22 Tier 4, *n* = 4 Tier 5) from 14 countries.

**Results:**

All surveyed coaches used some form of wearable technology to individualize training procedures. The most frequently used parameters included heart rate-related data (88.5% of participants) and GPS-related data (87.7% of participants). On a 1–7 Likert scale, coaches reported a 4.5 ± 1.4 (“somewhat agree”) that wearable technology influences decision making. Current use of wearable technology showed a significant positive association with “perceived job relevance”, while the influence of wearable technology on decision-making in training procedures was positively associated with “output quality”. The future “intention to use” wearable technologies correlated positively with “perceived usefulness”.

**Conclusion:**

All coaches in this study used some wearable technology to individualize training procedures. Coaches “somewhat agree” that wearables have an effect on their decision-making. For wearable technology to effectively influence coaches’ decision-making during training, these technologies must provide high quality outputs and must be perceived as useful to increase future adoption. Coaches expressed a need for demonstrable results and they must perceive wearables as useful.

**Supplementary Information:**

The online version contains supplementary material available at 10.1186/s40798-025-00919-5.

## Key Points

1) All 130 coaches within this study used some wearable technology to individualize training, but coaches only “somewhat agree” that wearables influence decision-making.

2) Heart rate and GPS related parameters are among the most used parameters optained by wearables.

3) Wearable manufacturers must produce products with high output quality and which are perceived as useful.

## Background

High inter- and intra-individual variability in training responses necessitates individualized approaches to optimize outcomes. One way to achieve this is by using both laboratory-derived data (e.g., physiological tests to define training intensity zones) and frequent monitoring of various parameters to guide day-to-day training decisions. Since it is not feasible to implement lab testing on a daily basis, wearable technologies are increasingly viewed as valuable devices to guide training related decisions [1]. For example, heart rate and heart rate variability measured during exercise are frequently used to assess exercise intensity in various endurance sports [2], and GPS data help coaches to evaluate training load and plan future sessions in soccer [3]. Wearables have also been used in resting conditions to guide training decisions. For example, nightly heart rate variability measurement can guide adjustments in endurance training [4].

While various approaches are promising for individualizing training, limited research exists on how coaches effectively utilize data from wearables. For instance, 113 U.S. strength and conditioning coaches and *n* = 7 triathlon coaches were interviewed about wearables [5, 6]. Both studies reported that although coaches use technology to enhance performance, they are cautious about over-relying on data [5, 6]. Despite these insights, current research is limited by its focus on U.S. coaches and its reliance on non-structured qualitative interviews, which can introduce bias [7].

Therefore, it remains unclear how and to what extent coaches use wearable data, what influences their usage, and what improvements are needed for further adoption. The technology acceptance model (TAM) has been widely used in various sectors to evaluate the acceptance and adoption of new technologies and may minimize bias as compared to non-structured interviews. A recent preliminary study surveyed 36 German-speaking coaches of various performance levels with the TAM to evaluate their use of wearable technologies, the factors influencing this usage, their impact on training decisions, and the key considerations for facilitating the future adoption of wearable technology for individualized training [8]. This preliminary study provided valuable initial insights, indicating that factors such as “perceived output quality” and “result demonstrability” play a critical role in influencing coaches’ decision-making processes when using wearable technology [8]. However, these findings are limited due to the small sample size and restriction to German-speaking coaches. The current investigation therefore extends beyond these preliminary findings by utilizing a bigger sample, incorporating coaches from diverse performance levels and geographical backgrounds.

The aim of the present research was to investigate (i) the current adoption of wearable technologies by coaches across different countries, (ii) the factors influencing current usage, (iii) the influence of wearable data on decision-making regarding training procedures, and (iv) the factors that necessitate improvement to facilitate further adoption of wearable technology for individualized training.

## Methods

### Questionnaire and Variables

The survey included questions on respondents’ socio-demographics, their current use of wearable technologies for individualizing training, factors influencing this current use, and factors affecting future intentions to use wearable technology for training purposes. Socio-demographic questions covered age, sex, performance level of coached athletes, and sporting disciplines. The use of wearable technology to individualize training procedures focused on the type of data utilized (e.g., heart rate data, GPS data, sleep data, subjective data) and the frequency of usage (e.g., daily, weekly, yearly).

To assess the factors influencing both current use and future intention to use wearable technologies for individualized training, we utilized existing operationalizations of the constructs included in the TAM as an empirical framework. The TAM has been frequently used in evaluating the application of other technologies across different contexts [9]. We adapted the wording of existing questions to ensure they were relevant to our specific research context. To fine-tune the TAM to address our specific research questions, we included exploratory items regarding the following aspects: desire to acquire knowledge about wearable technology for individualizing training procedures, desire for guidelines on how to effectively use wearable technology for individualized training, and impact of wearable technologies on altering coaches’ decisions related to individualized training.

The developed questionnaire was used in a recent investigation among German speaking coaches [8] (*n* = 36), which indicated that factors such as “perceived output quality” and “result demonstrability” play a critical role in influencing coaches’ decision-making processes regarding wearable technology [8]. This questionnaire (in the English language) was used and respondents were asked to rate the provided statement on a 7-point Likert Scale [9]. The original item formulation and their adaptations for the current study (expressed as means, standard deviations and range), including scale characteristics such as measures of internal consistency, are provided in Supplementary Table S1. Cronbach’s Alpha was applied to scales with more than two items, while item correlation was used to evaluate scale consistency for scales consisting of only two items [9]. Cronbach´s alpha was interpreted as excellent (≥ 0.9), good (> 0.8), acceptable (> 0.7), questionable (> 0.6), poor (> 0.5), and unacceptable (< 0.5) [10]. The reformulated questionnaire could not be assessed for its latent structure (linking the answers to questionnaire items with underlying, unobservable constructs) due to small sample size. The final sample consisted of coaches up to the elite level, meaning gathering a sufficiently large sample to assess scale integrity via structural equation modeling across all items was not feasible.

### Data Collection

The investigation was performed in accordance with the Declaration of Helsinki, except for pre-registration in a public repository. All procedures were approved by the ethical committee of Exercise Science & Training of the Faculty of Human Sciences of the University of Würzburg (EV2025/3-0304).

The survey was conducted online between December 2023 and April 2024 via the platform SoSciSurvey (SoSci Survey GmbH, Munich, Germany). Participants were recruited via various internet platforms, including social media and personal connections of the authors to coaches.

### Statistical Analysis

Descriptive analyses were conducted for relevant variables, including age and performance level of coaches (classified based on a recent framework [11]), use of wearable technology, and the influence of wearable technology on decision-making. Based on our previous study [8], the current investigation defines three main variables of interest: (a) the current use of wearable technology, (b) the influence of wearable technology on decision-making regarding individualized training procedures, and (c) the future intention to use wearable technology.

We also explored which factors influenced the three main outcomes using a multiple linear regression. Drawing on the findings from our pilot study [8] involving German coaches, we assumed that the influence of wearable technology data on coaches’ decision-making would be particularly related to its estimated “output quality” and “result demonstrability”. In contrast, the general use of wearable technology was expected to be influenced by social norms, job relevance, and perceived usefulness. Furthermore, we hypothesized that the intention to use wearable technology in the future would be influenced by the need for more guidelines, perceived usefulness, output quality, result demonstrability, and coaches’ willingness to acquire more knowledge about training individualization and wearable technology [8].

Following the hypotheses discussed above, a multiple, linear regression analysis was conducted for each of the three outcomes of interest. Within the regression models, both the independent variables (IVs) and dependent variables (DVs; outcome of interest) represent the mean item score of their respective scales resulting in continuously scaled variables. IVs were those scales that showed significant relationships with the respective DV in the initial sample. Notably, comparing the preliminary results of the pilot study [8] in a small German sample with the original TAM [12], we observed substantial differences in the intercorrelation across scales. To address this, regression analyses initially included “ease of use” and “perceived usefulness” (as outlined in the original TAM [12]) as covariates, before adding those IVs identified as most promising from our preceding results. As several IVs were highly correlated, these were introduced into the models in a step-wise fashion (see Table 2 for an overview). This approach allowed us to track any reduction in an IV’s predictive power (indicated by changes in levels of significance/t-values) due to multicollinearity, which may have otherwise obscured true relationships between the DVs and IVs. Within each final regression model, all effects were Bonferroni adjusted (if the model included four IVs, then *p*-values were adjusted for **five** tests, **also considering the “intercept” term**). Our statistical modelling approach integrated two primary sources of information: (a) the original TAM, which identified *perceived ease of use* and *perceived usefulness* as central predictors, and (b) findings from our pilot investigation, which suggested that the interrelationships among certain variables of interest deviated markedly from those posited by the original TAM. To account for both theoretical and empirical insights, we structured our models to first include the core TAM variables, followed by the stepwise addition of variables informed by our pilot data. This sequential modelling strategy enabled us to isolate the unique contribution of the newly considered variables beyond the explanatory power of the original TAM constructs. Adjusted *p*-values smaller than 0.05 were considered significant. All statistical analyses were performed using R (Version 2025.05.1).

We conducted a post hoc power analysis using GPower 3.1, selecting the “exact” test family and the “linear multiple regression: random model” test. This test assesses the power to detect a significant relationship between the full set of predictors and the dependent variable. To accurately reflect intercorrelations among predictors, we derived effect sizes based on the full correlation matrix of predictor and outcome variables. The correlations used for this calculation can be reviewed in the supplementary files.

## Results

### Participants

A total of 130 coaches from 14 countries (male: 119; female: 9; not indicated: 2; mean age: 42 ± 11 years) completed the questionnaire. Coaches originated from Italy (64%), the UK (11.4%), Switzerland (8.5%), Belgium (4.6%) Germany (3%) Sweden (2.3%), Austria (0.77%), Greece (0.77%), Iran (0.77%), the Netherlands (0.77%), Norway (0.77%), Spain (0.77%), the United States (0.77%), and Poland (0.77%). According to a classification framework [11], coaches indicated their athletes’ performance level as follows: level 1 (recreationally active, *n* = 5), level 2 (trained/developmental, *n* = 47), level 3 (highly trained/national level, *n* = 52), level 4 (elite/international level, *n* = 22), and level 5 (world class, *n* = 4).

The main sports in which the coaches were active included triathlon (49.00%), running (17.70%), cycling (6.90%), track and field (6.15%) swimming (6.15%), cross-country skiing (2.31%), rowing (0.77%), alpine skiing (0.77%), golf (0.77%), handball (0.77%), kayak (1.50%), football (0.77%), short track speed skating (0.77%) and hockey (0.77%). Five percent did not clearly indicate their main sport or listed multiple sports as their primary focus.

Our power analysis revealed a power of > 0.99 for all three regression models, suggesting that our sample size was adequate to detect meaningful effects.

### Use of Wearable Technologies and their self-reported Effect on Individualizing Training Procedures

Coaches utilized various types of data collected through wearable technologies, including heart rate data (e.g., time spent in heart rate zones, *n* = 115 out of 130), GPS-based data (e.g., distance covered during training, *n* = 114 out of 130), sleep-related data (e.g., sleep duration, *n* = 59 out of 130), heart rate variability parameters (*n* = 61 out of 130), and subjective parameters (*n* = 57 out of 130).

On a 1–7 Likert scale, coaches reported that they use wearable technologies for their daily (mean: 5.6 ± 1.4), weekly (mean: 6.0 ± 1.2), and annual planning (mean: 5.6 ± 1.6) of individualized training. Additionally, wearable technology influencing decisions to alter training strategies was reported as 4.5 ± 1.4.

### Factors Affecting Current and Future Use of Wearable Technologies To Individualize Training Procedures

Descriptive statistics, including Cronbach’s alpha, Pearson correlation, mean, and range for items included in the regression analysis, are presented in the Supplementary Table S1. Most of the scales used in the analyses showed acceptable (Cronbach´s alpha > 0.7) or good (Cronbach´s alpha > 0.8) internal consistency. The lowest score was found for the scale “willingness to learn more about sensor technology” (Cronbach´s alpha 0.54). Notably, this scale was the only one employing dichotomous items and was designed to encompass heterogeneous facets (e.g., a coach interested in GPS data may not share the same interest in sleep-related data, yet both items assess willingness to learn about technology).

Tables 1, 2 and 3 show the progression t-values for each variable across regression models which are further discussed in the following sections.

Further, model characteristics and their change following the inclusion of more predictors are shown to provide readers with a sense of effect sizes and multicollinearity.

**Table 1:** Progression of t-values and model characteristics for regressions on being “influenced by sensor technology”. VIF = variance inflation factor

Model Step		Model 1	Model 2	Model 3	Model 4
Coefficient t-values	Ease of use	2.27	0.636	-1.39	-1.54
	Perceived Usefulness	-	4.21	3.35	3.13
	Output Quality	-	-	4.43	3.73
	Result Demonstrability	-	-	-	1.09
Model Diagnostics	R²	0.042	0.17	0.292	0.299
	Adjusted R²	0.034	0.156	0.273	0.275
	VIF	-	1.17	1.397	1.493
	Δ R²	-	0.128	0.122	0.007
	Δ Adjusted R²	-	0.122	0.118	0.001
	Δ VIF	-	-	0.227	0.096

**Table 2:** Progression of t-values and model characteristics for regressions on “current use” of sensor technology. VIF = variance inflation factor

Model Step		Model 1	Model 2	Model 3	Model 4
Coefficient t-values	Ease of use	4.85	2.69	2.26	1.45
	Perceived Usefulness	-	6.8	6.01	3.23
	Subjective Norm	-	-	1.96	1.25
	Job Relevance	-	-	-	5.56
Model Diagnostics	R²	0.168	0.407	0.426	0.55
	Adjusted R²	0.161	0.397	0.411	0.534
	VIF	-	1.17	1.24	1.468
	Δ R²	-	0.239	0.019	0.123
	Δ Adjusted R²	-	0.236	0.014	0.122
	Δ VIF	-	-	0.07	0.228

**Table 3:** Progression of t-values and model characteristics for regressions on “intention to use” sensor technology. VIF = variance inflation factor

Model Step		Model 1	Model 2	Model 3	Model 4	Model 5
Coefficient t-values	Ease of use	2.87	0.56	-0.88	-0.65	-0.73
	Perceived Usefulness	-	6.73	5.95	5.81	5.43
	Result Demonstrability	-	-	4.49	3.85	3.95
	Learning about Tech	-	-	-	2.22	1.51
	Wish for Guideline	-	-	-	-	2.61
Model Diagnostics	R²	0.066	0.33	0.431	0.454	0.486
	Adjusted R²	0.058	0.318	0.416	0.435	0.463
	VIF	-	1.17	1.279	1.263	1.262
	Δ R²	-	0.264	0.101	0.024	0.031
	Δ Adjusted R²	-	0.26	0.097	0.02	0.028
	Δ VIF	-	-	0.108	-0.016	-0.001

### Current Use of Wearable Technology To Individualize Training Procedures

The current use of wearable technology to individualize training procedures was found to be closely associated with its “ease of use” (Table 1, Model 1). Part of this relationship can be attributed to “perceived usefulness”, as the inclusion of this variable in the model significantly reduced the predictive power of “ease of use” (Model 2). When subjective norms and job relevance were added to the model (Models 3 and 4), only “job relevance” and “perceived usefulness” retained a significant relationship with current use (Table 1).

Table 4 presents the results of the regression analysis for the final model, with the current use of wearable technology as the dependent variable. To check whether results were biased by a few coaches or a subsample of coaches who had unreasonable leverage on the data, we conducted assumption checks on the statistical assumptions of multiple regression analyses and recomputed the final model after exclusion of data with large leverage as assessed by Cook’s distances (see supplementary files).

**Table 4:** Results of the regression analysis of the final model with current use of wearable technology as dependent variable. After exclusion of coaches with large leverage on the data, significant effect sizes increased considerably for both initially significant effects. P (adj.) reflects Bonferroni adjusted p-values based on the number of terms in the final model (including the intercept)

Final Regression Model: Current Use										
Predictor	Unstandardized Estimate	SEM	95% CI (low)	95% CI (high)	*t*	*df*	*p*	*p* (adj.)	ϐ	partial η²
(Intercept)	5.767	0.076	5.617	5.918	76.089	113	0.000	0.000		
Ease of Use	0.123	0.085	-0.045	0.291	1.452	113	0.149	0.746	0.103	0.168
Perceived Usefulness	0.316	0.098	0.122	0.511	3.230	113	0.002	0.008	0.263	0.239
Subjective Norm	0.107	0.086	-0.063	0.276	1.246	113	0.215	1.000	0.088	0.019
Job Relevance	0.548	0.099	0.353	0.744	5.557	113	0.000	0.000	0.455	0.123
Final Regression Model: Current Use (without participants who had large leverage on the data)										
(Intercept)	5.945	0.057	5.832	6.058	104.396	104	0.000	0.000		
Ease of Use	0.082	0.065	-0.047	0.211	1.256	104	0.212	1.000	0.085	0.185
Perceived Usefulness	0.244	0.073	0.100	0.389	3.364	104	0.001	0.005	0.253	0.251
Subjective Norm	0.105	0.063	-0.019	0.230	1.677	104	0.097	0.483	0.109	0.030
Job Relevance	0.509	0.073	0.363	0.655	6.928	104	0.000	0.000	0.528	0.169

### Influence of Wearable Technology on Decision Making Regarding Individualization of Training Procedures

“Ease of use” was found to significantly correlate with the influence of wearable technology on decision-making (Table 2, Model 1). This relationship is further explained by “perceived usefulness” (Table 2, Model 2). When “output quality” (Table 2, Model 3) and “result demonstrability” (Table 2, Model 4) were included in the model, only “output quality” and “perceived usefulness” retained significant relationships.

Table 5 presents the results of the regression analysis for the final model, with the influence of wearables on decision making the dependent variable. To check whether results were biased by a few coaches or a subsample of coaches who had unreasonable leverage on the data, we conducted assumption checks on the statistical assumptions of multiple regression analyses and recomputed the final model after exclusion of data with large leverage as assessed by cook’s distances (see supplementary files).

**Table 5:** Results of the regression analysis of the final model with the influence of wearables on decision making as the dependent variable. Exclusion of highly impactful data lead to small decreases in effect sizes, indicating initial overestimation of effects due to the leverage of a few coaches on the data

Final Regression Model: Influence of Tech												
Predictor	Unstandadrdized Estimate	SEM	95% CI (low)	95% CI (high)	*t*	*df*	*p*	*p* (adj.)	ϐ	partial η²		
(Intercept)	4.516	0.108	4.301	4.731	41.662	113	0.000	0.000				
Ease of Use	-0.203	0.132	-0.465	0.059	-1.537	113	0.127	0.635	-0.148	0.042		
Perceived Usefulness	0.383	0.122	0.140	0.626	3.128	113	0.002	0.011	0.278	0.128		
Output Quality	0.530	0.142	0.249	0.812	3.730	113	0.000	0.002	0.384	0.122		
Result Demonstrability	0.144	0.133	-0.118	0.407	1.088	113	0.279	1.000	0.104	0.007		
Final Regression Model: Influence of Tech (without participants who had large leverage on the data)												
(Intercept)	4.591	0.102	4.388	4.794	44.800	105	0.000	0.000				
Ease of Use	-0.218	0.125	-0.467	0.031	-1.736	105	0.085	0.427	-0.164	0.051		
Perceived Usefulness	0.329	0.122	0.087	0.571	2.691	105	0.008	0.041	0.248	0.142		
Output Quality	0.520	0.133	0.256	0.784	3.907	105	0.000	0.001	0.393	0.147		
Result Demonstrability	0.262	0.128	0.008	0.516	2.046	105	0.043	0.216	0.198	0.025		

### Intention To Use Wearable Technology in the Future

The intention to use wearable technology in the future was generally associated with “ease of use” (Table 3, Model 1). However, when “perceived usefulness” was included in the model, “ease of use” was no longer correlated with the “intention to use wearable technology in the future” (Table 3, Model 2), indicating that “perceived usefulness” largely accounts for the overlap between “ease of use” and the “intention to use”. Further incorporating “result demonstrability” (Table 3, Model 3), “willingness to learn about wearable technology” (Table 3, Model 4), and the “desire for guidelines” (Table 3, Model 5) revealed that the intention to use wearable technology in the future is primarily related to “perceived usefulness,” the “wish for guidelines” (marginally significant after Bonferroni correction), and “result demonstrability”. Importantly, the effect of “wish for guidelines” seems to be driven by the impact of a few cases, as its predictive value plummeted considerably after excluding coaches with large leverage on the data from the analysis. Table 6 presents the results of the regression analysis for the final model, with the current use of wearable technology as the dependent variable. To check whether results were biased by a few coaches or a subsample of coaches who had unreasonable leverage on the data, we conducted assumption checks on the statistical assumptions of multiple regression analyses and recomputed the final model after exclusion of data with large leverage as assessed by Cook’s distances (see supplementary files).

**Table 6:** Results of the regression analysis of the final model with intention to use wearable technologies as dependent variable. Exclusion of coaches who showed large impact on the result lead to considerable changes regarding “wish for guidelines” rendering it insignificant

Final Regression Model: Intention to Use										
Predictor	Unstandandardized Estimate	SEM	95% CI (low)	95% CI (high)	*t*	*df*	*p*	*p* (adj.)	ϐ	partial η²
(Intercept)	5.754	0.088	5.579	5.928	65.376	112	0.000	0.000		
Ease of Use	-0.071	0.097	-0.263	0.122	-0.728	112	0.468	1.000	-0.057	0.066
Perceived Usefulness	0.524	0.097	0.333	0.715	5.430	112	0.000	0.000	0.418	0.264
Result Demonstrability	0.395	0.100	0.197	0.593	3.954	112	0.000	0.001	0.314	0.101
Learning about Tech	0.148	0.098	-0.046	0.342	1.512	112	0.133	0.801	0.111	0.024
Wish for Guidelines	0.298	0.114	0.072	0.524	2.609	112	0.010	0.062	0.189	0.031
Final Regression Model: Intention to Use (without participants who had large leverage on the data)										
(Intercept)	6.009	0.067	5.875	6.143	89.131	106	0.000	0.000	0.000	
Ease of Use	-0.097	0.080	-0.255	0.061	-1.219	106	0.226	1.000	-0.103	0.043
Perceived Usefulness	0.487	0.076	0.338	0.637	6.457	106	0.000	0.000	0.519	0.308
Result Demonstrability	0.269	0.079	0.112	0.425	3.404	106	0.001	0.006	0.286	0.081
Learning about Tech	0.083	0.073	-0.061	0.227	1.143	106	0.256	1.000	0.088	0.010
Wish for Guidelines	0.080	0.072	-0.062	0.222	1.115	106	0.267	1.000	0.085	0.006

## Discussion

We aimed to investigate (i) whether coaches currently use wearable technologies, (ii) which factors influence current usage, (iii) whether data provided by wearables impact coaches’ decision-making regarding training procedures, and (iv) to evaluate the factors that need improvement to facilitate the intention to use wearable technology for individualized training by coaches.

Our main findings were as follows:

All 130 coaches surveyed used some form of wearable technology to individualize training procedures. Heart rate data (e.g., time spent in heart rate zones; 88.5% of coaches) was the most used parameter, followed by GPS-derived parameters ( 87.7% of coaches), heart rate variability (46.9% of coaches), and sleep-related parameters (45.4% of coaches). On a 1–7 Likert scale, coaches reported using wearable technologies for their daily (mean: 5.6 ± 1.4), weekly (mean: 6.0 ± 1.2), and annual (mean: 5.6 ± 1.6) planning of individualized training. On a 1–7 Likert scale, coaches’ average agreement was 4.5 ± 1.4 (“somewhat agree”) to the statement that wearable technology influences decision making.

The current use of wearable technology primarily correlated with “perceived job relevance” (explaining 16.9% of variance in current use), which was closely related to “perceived usefulness” (explaining 25.1% of variance in current use). The extent to which coaches’ decision-making was influenced by data obtained from wearable technology was mainly related to “output quality” (explaining 14.7% of variance in the extent of influence) and “perceived usefulness” (explaining 14.2% of variance in the extent of influence). The intention to use wearable technology in the future was primarily associated with “perceived usefulness” (explaining 30.8% of variance in the intention to use) and “result demonstrability” (explaining 8.1% of variance in the intention to use).

### Current Use of Wearable Technology and their Influence To Individualize Training Procedures

Our analysis indicates that the current use of wearable technology was strongly correlated with “perceived ease of use”, though stepwise predictor introduction revealed that this relationship is largely mediated by “perceived usefulness”. Thus, the current use of wearable technology is more dependent on “perceived usefulness” than on “ease of use”. Although “ease of use” and “perceived usefulness” are strongly correlated, this does not imply causation. The current data do not clarify whether easier-to-use technology is perceived as more useful or if technology deemed useful becomes easier to use over time. Notably, coaches’ usage was unaffected by “social norms”, indicating that their decisions were based on personal assessments of usefulness.

While the use of wearable technology itself may provide useful information, evaluating whether such usage translates into actionable decisions is equally important. Our results indicate that all coaches investigated in this study used wearable technology, and they reported a score of 4.5 ± 1.4 (on a 1–7 scale, “somewhat agree”) for individualizing training procedures. The observed variability (SD of 1.4) suggests a degree of ambivalence regarding wearables’ impact in this context, which may stem from several factors. For example, factors influencing whether coaches individualize training procedures based on wearable data include “output quality” and “perceived usefulness”. As different coaches (with potentially different educational backgrounds) use different wearable technologies, this could influence the output quality a specific coach experiences during training. Our findings underscore the importance of “output quality” and “perceived usefulness” in promoting meaningful use of wearable technology. To support adoption, manufacturers should prioritize the development of devices that provide high quality data. At the same time, sports organizations might enhance the practical impact of wearables by offering targeted education and training for coaches to perceive them as useful.

Our findings are somewhat in-line with available literature, as it has been previously reported that strength and conditioning coaches and athletic trainers in the USA reported frustration about technology providing inaccurate data (similar to the “output quality” outcome in our study), and that knowing “what the data mean” (similar to the “perceived usefulness” outcome in our study) is a critical step when purchasing a wearable technology [5].

Despite the findings of our study and previous literature of coaches perceptions and requirements regarding wearable technologies, evidence shows that wearable technologies often generate data with inadequate output quality, particularly concerning reliability and validity [13, 14]. Our results highlight the need for manufacturers to release products with consistently high output quality. This is in line with findings among recreational athletes who also indicated that high output quality is an important factor influencing the use of wearables [15, 16] and since it was shown that devices which provide high quality data can be used to inform accurately assessing relevant parameters can be used to inform decision-making of coaches [16, 17].

In the context of output quality, it is interesting to note that 115 out of 130 coaches indicated they used parameters related to heart rate (i.e., time spent in different intensity zones). As this is one of the most monitored parameters, it seems important to mention, that HR zones should be individualized based on laboratory assessments of metabolic thresholds, as HR zones defined as percentages of maximal heart rate or maximal oxygen uptake as typically done in wearables, for example, poorly conform to exercise intensity domains, and thus do not adequately control for the intended metabolic stimulus and subsequent training adaptations [18].

### Future Intention To Use Wearable Technology

The intention to increase the use of wearable technology in the future was strongly related to “perceived usefulness,” and “result demonstrability.” For wearable technologies to be effectively implemented in sports practice for individualized training, wearable manufacturers, scientists, and authorities responsible for developing guidelines (e.g., governing bodies of various sports) must address these factors.

Arguably, enhancing the “perceived usefulness” of wearable technologies could be achieved by providing scientific evidence of their effectiveness and clearly linking measured parameters to intended outcomes. Monitoring and data collection alone are insufficient, as the data must be translated into actionable insights [15]. For example, evidence suggests that accurate measurement and interpretation of resting heart rate variability (HRV) can offer valuable training intensity recommendations for endurance athletes [19]. Research has demonstrated that incorporating HRV monitoring in endurance training leads to greater improvements in submaximal performance parameters compared to preplanned training protocols, emphasizing the value of HRV monitoring in guiding training [19]. However, for other wearable technologies, the evidence that monitoring parameters leads to positive training outcomes, such as enhanced performance or reduced injury risk, is often insufficient. For instance, some research-grade wearable devices claim to assess lactate levels in sweat. However, there is a debate around the agreement between sweat and blood lactate levels [20]. Consequently, the usefulness of measuring lactate in sweat currently remains limited in practical sports applications. Providing robust evidence that monitored parameters can be transformed into actionable insights could significantly enhance “perceived usefulness” and, consequently, the future intention to use wearable technologies.

## Strength, Limitations and Future Research

A strength of this study is that it builds on prior research while expanding its scope to 130 international coaches from 14 countries, representing various performance levels, including elite coaches. Moreover, the study is grounded in the established TAM, which provides a robust theoretical framework. A potential study limitation is that the majority of participants were recruited from Europe (especially Italy), were male and primarily represented endurance-based sports (and primary triathlon coaches). Further investigations involving more coaches from other continents, a broader range of sports and investigating their educational backgrounds would provide a more comprehensive understanding of the current use of wearable technologies, their future adoption, and their influence on decision-making in training procedures. Additionally, it could be possible that coaches from different sports adopt wearables differently and that different sports cultures and technological infrastructures affect perceived usefulness, ease of use, and output quality assessments. Future research should be performed within different sports to further elucidate the technology adoption of wearables in different sports disciplines, performance levels and and include more female coaches.

Furthermore, as new wearables measuring a variety of parameters (e.g. biomechanical variables, tissue loadings, blood pressure) are becoming available, assessing the use of such devices by coaches and athletes could be an avenue for future research.

## Conclusions

All coaches in this study used some form of wearable technology to individualize training procedures. On a 1–7 Likert scale, coaches’ average agreement was 4.5 ± 1.4 (“somewhat agree”) to the statement that wearable technology influence decision making. This could imply that coaches prioritize high-quality data and if data are of low quality , they are unlikely to drive decisions, even if available. Several factors correlate with (i) the current use of wearable technology, (ii) the influence of wearable technology on coaches’ decisions to alter training procedures, and (iii) the future intention to use wearable technology for individualized training. Our investigation revealed that, in order for wearable technology to effectively influence coaches’ decision-making during training, these technologies must provide high output quality and must be perceived as useful. For the future, coaches expressed need for demonstrable results and they must perceive wearables as useful.

## Supplementary Information

Below is the link to the electronic supplementary material.

Supplementary Material 1

## Data Availability

Additional information can be found in the Supplementary Table S1, further inquiries can be directed to the corresponding author.

## Abbreviations

CI: Confidence Interval
Df: Degrees of Freedom
DV: Dependent Variable
GPS: Global Positioning System
HR: Heart Rate
HRV: Heart Rate Variability
IV: Independent Variable
mM: Millimol
SE: Standard Error
TAM: Technology Acceptance Model
