# Supplementary material for: Global Insights on Wearable Technology Adoption by Coaches: Determinants of Current Use, Decision Making, and Future Intention To Use
Source: Sports Med Open. 2025 Nov 21;11:131. doi: 10.1186/s40798-025-00919-5 (PMC12638525; doi:10.1186/s40798-025-00919-5)

**Table S1: Descriptive statistics, including Cronbach’s alpha, Pearson correlation, mean, and range for items included in the regression analysis.**

| **Scale/Item** | **Internal Consistency (Cronbachs Alpha or Pearson correlation for scales with <3 items)** | **Mean** | **Standard Deviation** | **Range (Sum)** |
| --- | --- | --- | --- | --- |
| Ease of use | 0.88 | 5.31 | 1.12 | 6-42 |
| ... is clear and understandable |  | 5.68 | 1.17 | 1-7 |
| ... does not require significant mental effort from me. |  | 5.17 | 1.61 | 1-7 |
| ... is easy to use. |  | 5.53 | 1.29 | 1-7 |
| Data collection using various wearable technologies to assess/analyse data is simple |  | 5.53 | 1.31 | 1-7 |
| Data analysis using various wearable technologies to assess/analyse data is simple. |  | 5.14 | 1.33 | 1-7 |
| Data interpretation using various wearable technologies to assess/analyse data is simple. |  | 4.83 | 1.63 | 1-7 |
| Informed about training | 0.88 | 5.62 | 1.12 | 4-28 |
| ... and performance improvements. |  | 5.86 | 1.25 | 1-7 |
| ... and physiological adaptations. |  | 5.73 | 1.22 | 1-7 |
| ... and recovery. |  | 5.76 | 1.19 | 1-7 |
| ... and injuries. |  | 5.11 | 1.54 | 1-7 |
| Informed Technology | 0.83 | 5.67 | 1.03 | 2-14 |
| ... how various wearable technologies to assess/analyse data can assist me in individualizing training |  | 5.65 | 1.18 | 1-7 |
| ... the (usage) possibilities and limitations of various wearable technologies to assess/analyse data for individualizing training |  | 5.69 | 1.04 | 1-7 |
| Subjective Norm | 0.94 | 4.57 | 1.39 | 2-14 |
| People who influence my behavior think that I should use various wearable technologies to assess/analyse data for individualizing training. |  | 4.58 | 1.40 | 1-7 |
| People who are important to me think that I should use various wearable technologies to assess/analyse data for individualizing training. |  | 4.56 | 1.45 | 1-7 |
| Wish for Guidelines | NA | 4.71 | 1.67 | 1-7 |
| I would wish that my club/association would provide me with a guideline for the use of wearable technologies to assess/analyse data. |  | 4.71 | 1.67 | 1-7 |
| Voluntariness | 0.54 | 5.13 | 1.26 | 3-21 |
| The use of wearable technologies to assess/analyse data to individualize training is voluntary |  | 5.74 | 1.40 | 1-7 |
| My supervisors/responsibe person at the club does not require me to use wearable technologies to assess/analyse data to individualize training |  | 5.04 | 1.73 | 1-7 |
| Although it might be helpful, using wearable technologies to assess/analyse data to individualize training is certainly not compulsatory in my job |  | 4.62 | 2.03 | 1-7 |
| Image | 0.81 | 4.86 | 1.33 | 3-21 |
| Coaches in my league who use wearable technologies to assess/analyse data to individualize training have more prestige than those who do not |  | 4.90 | 1.53 | 1-7 |
| Coaches in my league who use wearable technologies to assess/analyse data to individualize training have a high profile |  | 4.93 | 1.44 | 1-7 |
| Using monitoring technologies to assess/analyse data to individualize training is a status symbol among coaches |  | 4.75 | 1.70 | 1-7 |
| Job Relevance | 0.76 | 6.12 | 1.01 | 2-14 |
| ... is important for my coaching work. |  | 6.13 | 1.07 | 1-7 |
| ... is relevant to my coaching work. |  | 6.12 | 1.09 | 1-7 |
| Current use | 0.81 | 5.77 | 1.2 | 3-21 |
| ... in daily planning for individualizing training. |  | 5.64 | 1.42 | 1-7 |
| ... in weekly planning for individualizing training. |  | 6.04 | 1.19 | 1-7 |
| ... in annual planning for individualizing training. |  | 5.64 | 1.62 | 1-7 |
| Output Quality | 0.64 | 5.12 | 1.36 | 2-14 |
| The reliability and validity of the wearable technologies to assess/analyse data is high. |  | 5.26 | 1.38 | 1-7 |
| I have no problem with the reliability and validity of wearable technologies to assess/analyse data. |  | 4.97 | 1.61 | 1-7 |
| Result demonstrability | 0.6 | 5.18 | 1.01 | 4-28 |
| I have no difficulty telling others about the results of wearable technologies to assess/analyse data for individualizing training. |  | 5.88 | 1.24 | 1-7 |
| I believe I could communicate the consequences of wearable technologies to assess/analyse data for individualization of training to others. |  | 5.82 | 1.31 | 1-7 |
| The results of using wearable technologies to assess/analyse data to individualize training processes are apparent to me. |  | 5.62 | 1.35 | 1-7 |
| I would have difficulty explaining why using wearable technologies to assess/analyse data for individualizing training may or may not be beneficial. |  | 3.39 | 1.96 | 1-7 |
| Influence of Tech | 0.86 | 4.53 | 1.38 | 4-28 |
| I am strongly influenced by wearable technologies to assess/analyse data when individualizing training. |  | 4.70 | 1.55 | 1-7 |
| I alter training procedures when wearable technologies to assess/analyse data suggests that I should train less intensively and/or for a shorter duration than I had planned. |  | 4.49 | 1.68 | 1-7 |
| I alter my planned training schedule if I have wearable technologies to assess/analyse data that suggests it. |  | 4.49 | 1.63 | 1-7 |
| I alter training procedures when wearable technologies to assess/analyse data suggests that I should train more intensively and/or for a longer duration than I had planned. |  | 4.43 | 1.73 | 1-7 |
| Intention to use | 0.75 | 5.85 | 1.26 | 2-14 |
| ... I intend to use such wearable technologies to assess/analyse data. |  | 5.86 | 1.34 | 1-7 |
| ... I predict that I would use it to individualize training. |  | 5.84 | 1.35 | 1-7 |
| Learning about training | 0.93 | 6.26 | 0.99 | 3-21 |
| ... and performance improvement. |  | 6.27 | 1.11 | 1-7 |
| ... and recovery management. |  | 6.2**9** | 1.05 | 1-7 |
| ... and reducing the likelihood of injuries. |  | 6.22 | 1.05 | 1-7 |
| Learning about tech | 0.54 | 2.64 | 1.66 | 7-14 |
| ... learn more about the application of wearable technologies to assess/analyse data collect and analyse data. |  | 1.**70** | NA | 1-2 |
| ... use more wearable technologies to assess/analyse data than I currently do. |  | 1.4**2** | NA | 1-2 |
| ... incorporate and utilize more heart rate data than I currently do. |  | 1.2**9** | NA | 1-2 |
| ... collect and utilize more GPS-based data (distance covered during training, speed, etc.) than I currently do. |  | 1.2**3** | NA | 1-2 |
| ... collect and utilize more sleep-related data (e.g., sleep duration, bedtime, etc.) than I currently do. |  | 1.4**1** | NA | 1-2 |
| ... incorporate and utilize more heart rate variability data than I currently do. |  | 1.4**2** | NA | 1-2 |
| ... collect and utilize other data than I currently do. Please indicate which data you would like to use more |  | 1.1**9** | NA | 1-2 |
| Perceived usefulness | 0.84 | 6.17 | 0.86 | 4-28 |
| ... improves individualization of training |  | 6.24 | 0.97 | 1-7 |
| ... increases my productivity |  | 5.79 | 1.26 | 1-7 |
| ... allows me to more effectively individualize training |  | 6.3 | 1.07 | 1-7 |
| ... is useful for individualizing training |  | 6.36 | 0.84 | 1-7 |

Supplementary Figure F1: *Diagnostic plots for the final model with “Current Use” as dependent variable. Importantly, it should be noted that the final model (before and after exclusion of impactful data points) was indicated to feature non-normal residuals (Shapiro-Wilk Test: p < .001).*


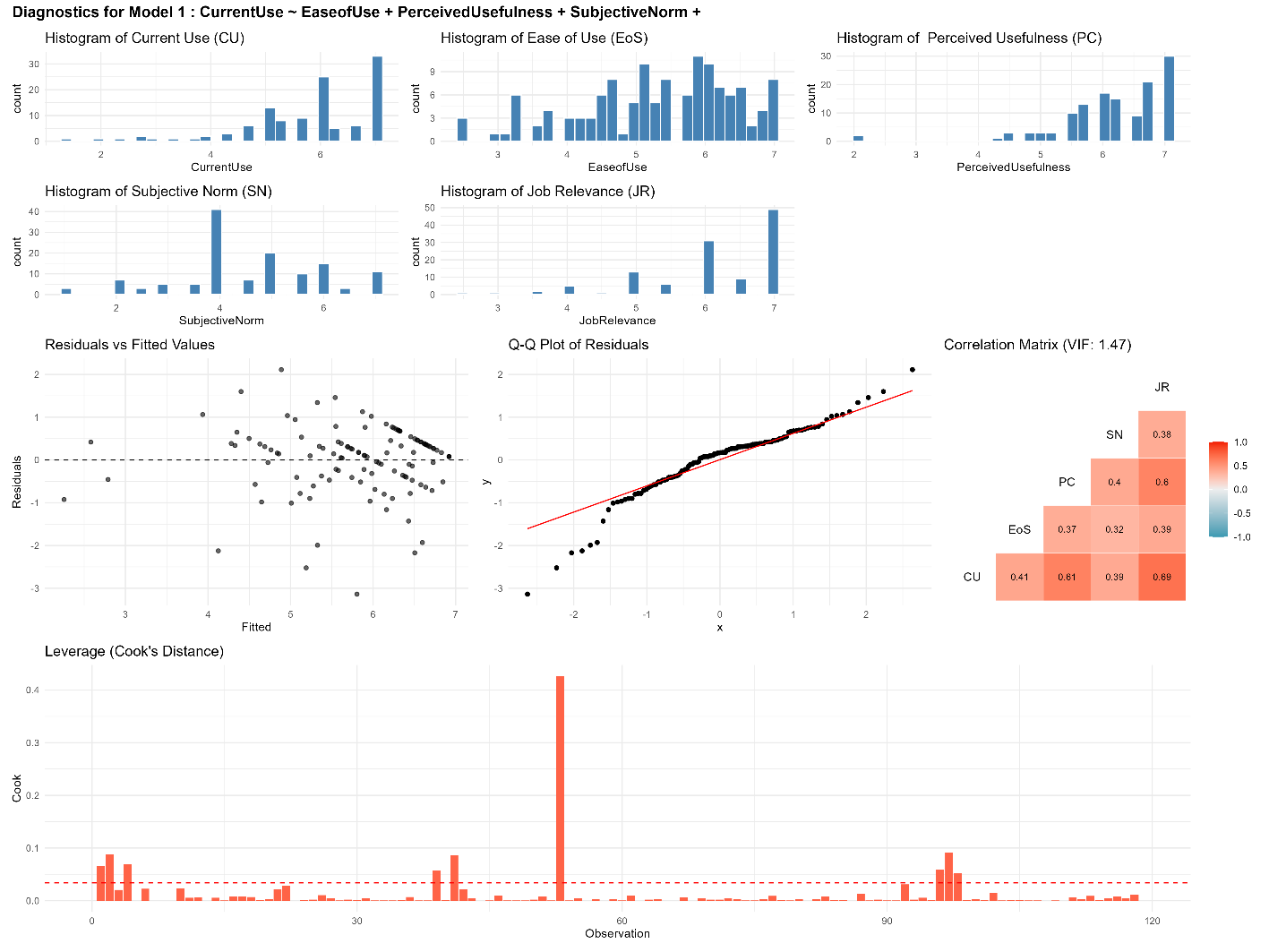


Supplementary Figure F1*. Diagnostic plots for the final model with “Influence by sensor technology” as dependent variable. Again, it should be noted that the final model (before and after exclusion of impactful data points) was indicated to feature non-normal residuals (Shapiro-Wilk Test: p < .001).*


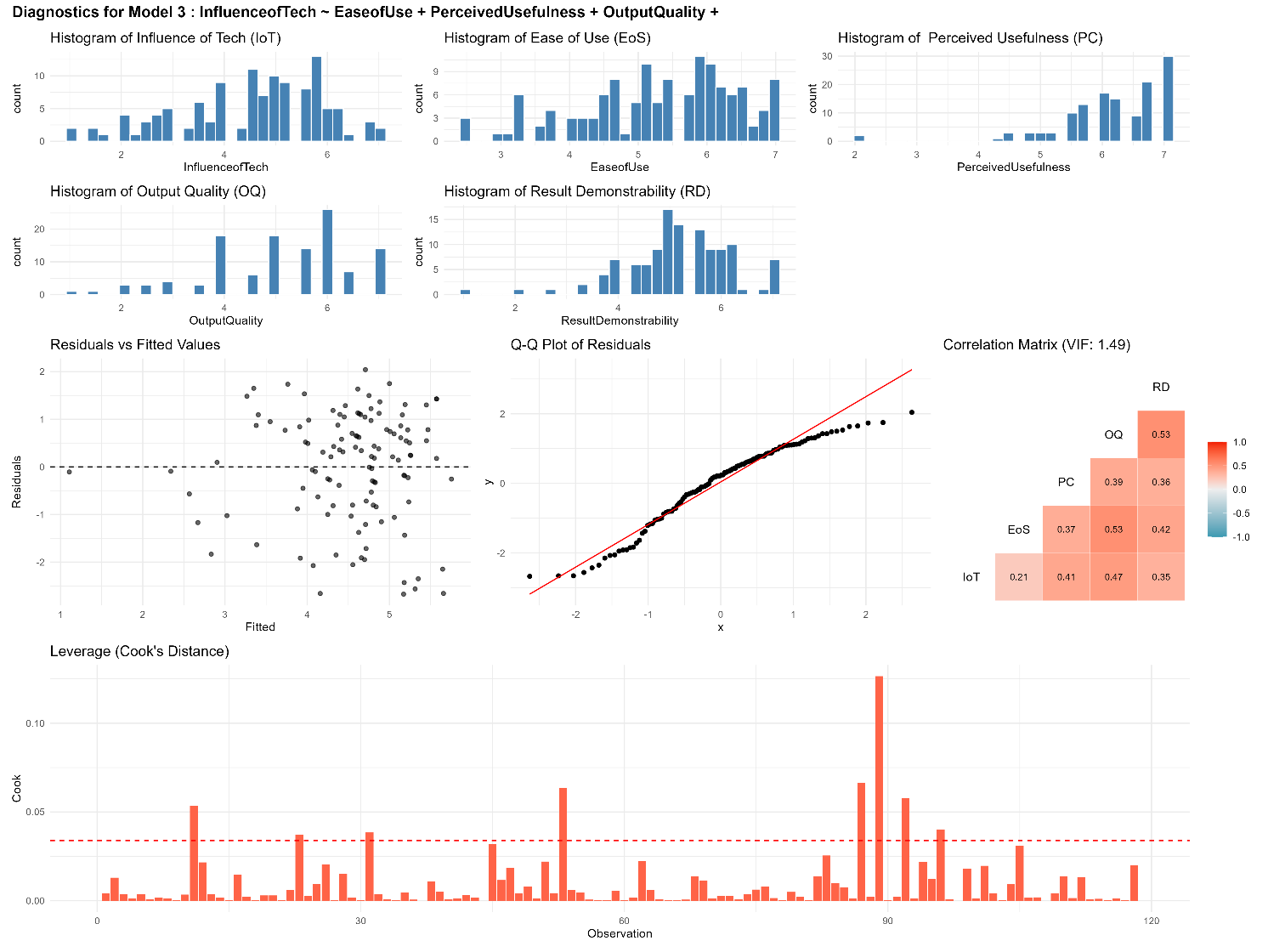


*Supplementary Figure F3. Diagnostic plots for the final model with “Intention to use” as dependent variable. Again, it should be noted that the final model (before and after exclusion of impactful data points) was indicated to feature non-normal residuals (Shapiro-Wilk Test: p < .001).*


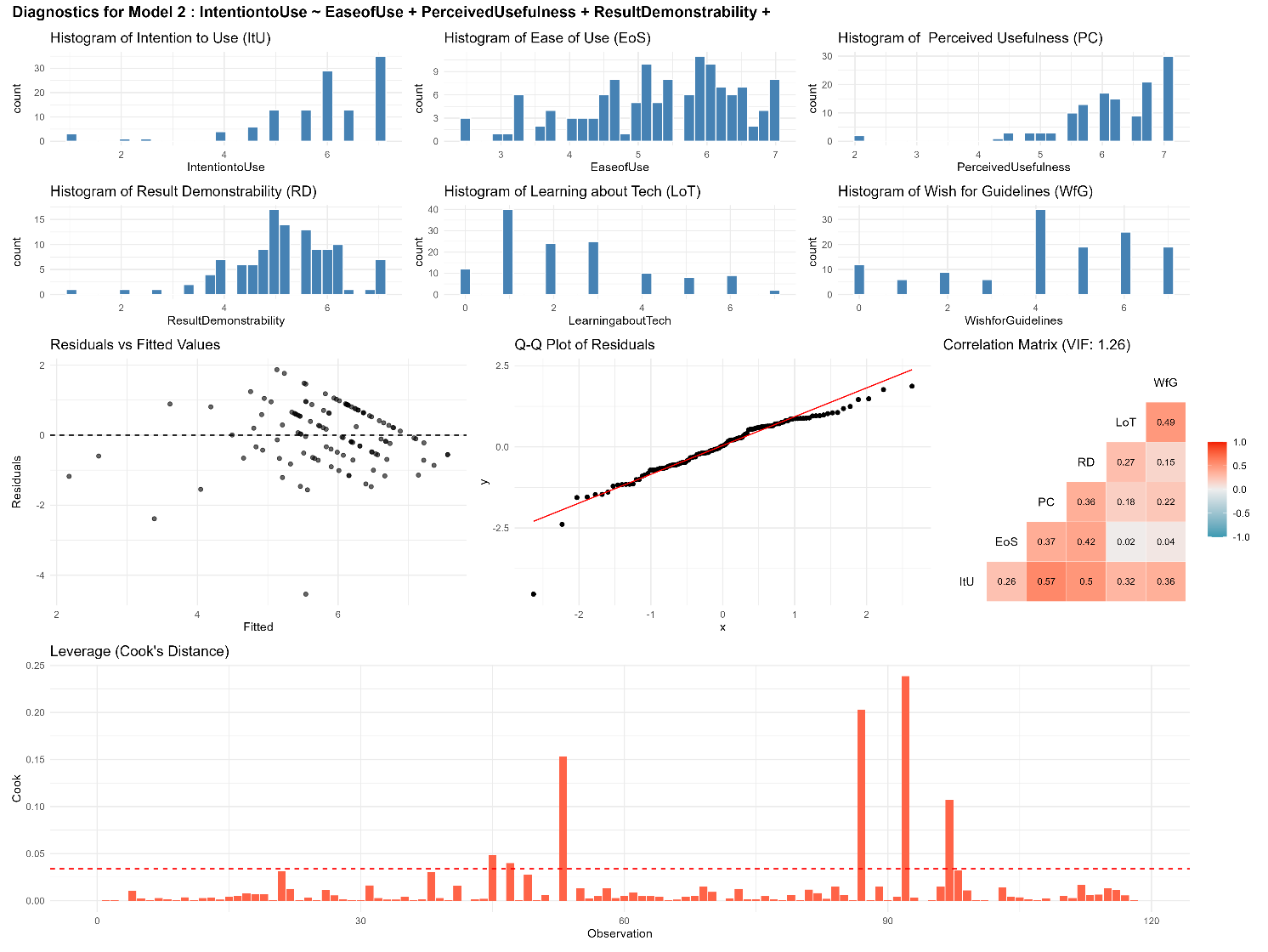

Supplement: Supplementary file 1 — Supplementary Material 1 [file 40798_2025_919_MOESM1_ESM.docx]
